# How teacher support shapes reading literacy among urban primary school pupils in China: evidence from achievement emotions and reading self-efficacy

**DOI:** 10.3389/fpsyg.2026.1909401

**Published:** 2026-10-01

**Authors:** Xue Feng, Wee Hoe Tan, Yue Xiang, Dahlia Janan, Ghayth Kamel Shaker Al-Shaibani

**Affiliations:** 1 School of Marxism, Capital Normal University, Beijing, China; 2 Faculty of Social Sciences and Liberal Arts, UCSI University, Kuala Lumpur, Malaysia; 3 School of Future Education, Qingdao Hengxing University of Science and Technology, Qingdao, China; 4 Faculty of Languages and Communication, Universiti Pendidikan Sultan Idris, Tanjong Malim, Malaysia; 5 Institute of Languages, UCSI University, Kuala Lumpur, Malaysia

**Keywords:** achievement emotions, primary school, quality education (SDG 4), reading literacy, self-efficacy, teacher support

## Abstract

**Background:**

Reading literacy is a fundamental determinant of academic achievement. However, the complex psychological mechanisms linking teacher support to reading literacy during the critical developmental transition of middle childhood remain insufficiently understood. This gap is particularly evident within the Confucian Heritage Culture (CHC) context, where teacher authority and social validation are highly prominent. Grounded in an integration of control-value theory and social cognitive theory, this study aims to investigate the parallel mediating roles of achievement emotions (an affective pathway) and reading self-efficacy (a cognitive-motivational pathway) in the relationship between perceived teacher support and reading literacy.

**Methods:**

A cross-sectional survey was conducted among 460 Grade 4 pupils (aged 9–10 years) from two public schools in Beijing, China. Data were collected using validated self-report questionnaires for psychological constructs and a Chinese adaptation of the PIRLS assessment for reading literacy, followed by analysis using covariance-based structural equation modeling (CB-SEM).

**Results:**

The structural model revealed that perceived teacher support significantly and positively predicted reading literacy (*β* = 0.446, *p* <0.001). Mediation analyses using bias-corrected bootstrapping demonstrated that achievement emotions significantly mediated the relationship between teacher support and reading literacy (*β* = 0.063, 95% CI [0.015, 0.115]). Conversely, reading self-efficacy did not exhibit a significant independent mediation effect when accounting for emotional factors. The total model explained 31.3% of the variance in reading literacy.

**Discussion:**

These findings highlight that teacher support enhances primary school pupils’ reading literacy primarily through emotional rather than cognitive-motivational pathways. The study extends existing literature by demonstrating that fostering emotionally supportive classroom environments and positive achievement emotions is a vital, yet often overlooked, pedagogical strategy for sustainable literacy development.

## 1 Introduction

### 1.1 Background and research questions

Reading literacy is widely recognized as a fundamental determinant of academic achievement, lifelong learning, and social participation. During the transition from lower to middle primary school, particularly around Grade 4, pupils move beyond basic decoding skills toward more advanced comprehension, inference and interpretation of texts (Barton et al., 2024). This developmental period requires students to engage with increasingly complex reading materials that demand higher levels of cognitive and emotional processing. Research consistently demonstrates that strong reading literacy is associated with better educational attainment and future socioeconomic outcomes. Conversely, pupils with persistent reading difficulties are more likely to experience academic underachievement and reduced educational opportunities (James et al., 2024). Although reading literacy is influenced by multiple factors, educational researchers continue to emphasize the importance of identifying mechanisms that support literacy development during middle childhood. Consequently, understanding the factors that promote reading literacy remains a central concern in contemporary educational research.

The challenge of reading literacy development is particularly significant during the middle primary school years. At this stage, pupils encounter a broader range of texts and are expected to demonstrate increasingly sophisticated comprehension skills. These expectations require learners to integrate prior knowledge, infer meanings and critically evaluate textual information. Empirical studies indicate that successful adaptation to these literacy demands contributes positively to academic performance across multiple subject areas (Hossain, 2024). However, not all pupils adapt equally well to these developmental expectations, resulting in substantial differences in reading outcomes. Furthermore, early literacy difficulties often persist into later educational stages if appropriate support mechanisms are not provided. Therefore, identifying effective contextual and psychological factors that facilitate reading literacy development is both theoretically and practically important.

Teacher support represents one of the most influential contextual factors within the classroom environment. Teacher support typically encompasses affective, instructional and evaluative forms of assistance that help students navigate academic challenges and sustain engagement in learning activities. Through daily interactions, teachers shape students’ perceptions of competence, belonging and academic expectations. Previous research consistently demonstrates that supportive teacher-student relationships are associated with higher levels of motivation, engagement and academic achievement (Granziera et al., 2022; Wang et al., 2022). Nevertheless, teacher support should not be viewed solely as an instructional resource because its influence extends beyond cognitive learning outcomes. In many educational settings, teacher support also serves as an important source of emotional reassurance and psychological security. Consequently, teacher support may represent a critical foundation for successful literacy development among primary school pupils.

The influence of teacher support may be particularly pronounced within the context of the Confucian Heritage Culture (CHC) in China. In CHC societies, teachers are traditionally regarded as respected authorities whose evaluations carry considerable social and academic significance. As a result, primary school pupils often rely heavily on teacher feedback and recognition when forming beliefs about learning and achievement. Existing studies suggest that external validation remains especially important during middle childhood because students are still developing stable self-concepts and learning identities (Ryan and Deci, 2023). However, the impact of teacher support cannot be fully explained through social influence alone because students also interpret and internalize these experiences through psychological processes. These psychological responses may determine whether supportive classroom experience ultimately translate into improved academic outcomes. Therefore, investigating the mechanisms through which teacher support affects reading literacy in necessary.

Achievement emotions constitute one important psychological pathway linking teacher support and reading literacy. According to Control-Value Theory (CVT), students develop specific emotional responses based on their perceptions of control over learning activities and the value they assign to academic tasks. Positive emotions such as enjoyment and pride tend to facilitate engagement and persistence, whereas negative emotions such as anxiety and boredom any hinder learning performance (Pekrun, 2024). Empirical evidence suggests that achievement emotions are closely associated with reading engagement and literacy outcomes across different educational contexts. Nevertheless, emotional experiences do not emerge independently but are strongly shaped by social interactions within the classroom environment. Supportive teacher behaviors may enhance students’ perceptions of control and value, thereby promoting more adaptive emotional experience. Consequently, achievement emotions may serve as an important affective mechanism through which teacher support contributes to reading literacy.

Reading self-efficacy represents a second psychological pathway through which teacher support may influence reading literacy. Within Social Cognitive Theory (SCT), self-efficacy reflects the confidence of an individual in successfully performing specific tasks and overcoming challenges. In literacy contexts, students with stronger reading self-efficacy are more likely to persist when confronted with difficult texts and to employ effective reading strategies (Song, 2024). Numerous studies have reported positive associations between self-efficacy beliefs and academic achievement across different learning domains. However, self-efficacy beliefs are particularly sensitive to external feedback during childhood because young learners often rely on significant adults to evaluate their own capabilities. Teacher encouragement and constructive guidance may therefore strengthen students’ confidence in their reading abilities. As a result, reading self-efficacy may function as an important cognitive-motivational mechanism connecting teacher support and reading literacy.

Although teacher support has consistently been associated with literacy achievement, less is known about the relative importance of emotional and cognitive-motivational pathways during middle childhood, particularly within Confucian Heritage Culture contexts. Existing studies have typically examined achievement emotions and self-efficacy independently, resulting in a fragmented understanding of the mechanisms underlying literacy development. While previous research supports the importance of both constructs, few studies have investigated their parallel mediating roles within a single theoretical framework. This limitation is especially evident among Chinese Grade 4 pupils, who occupy a critical developmental stage characterized by increasing academic demands and continued dependence on teacher support. Furthermore, little is known about whether emotional pathways or cognitive-motivational pathways exert stronger influence on reading literacy outcomes. Addressing this gap may contribute to a more comprehensive understanding of how classroom support shapes literacy development. Therefore, the present study shapes literacy development. Therefore, the present study integrates CVT and SCT to examine the parallel mediating roles of achievement emotions and reading efficacy in the relationship between perceived teacher support and reading literacy.

Consequently, this study focuses on Grade 4 pupils in an urban context of Beijing and investigates how perceived teacher support influences reading literacy through affective and cognitive-motivational mechanisms among urban Chinese primary school children. Specifically, the study examines both the direct relationship between teacher support and reading literacy and the indirect relationships mediated by achievement emotions and reading self-efficacy. This approach enables a more comprehensive evaluation of the psychological processes underlying literacy development. Based on the proposed theoretical framework, two research questions are formulated.

*RQ1*: In the context of Chinese primary education, does perceived teacher support significantly and positively predict students’ reading literacy?

*RQ2*: Do achievement emotions and reading self-efficacy act as parallel mediators in the relationship between perceived teacher support and reading literacy?

By addressing these questions, the study aims to contribute both theoretical and practical insights into the promotion of reading literacy among primary school pupils.

### 1.2 Literature review and theoretical model

#### 1.2.1 Teacher support and reading literacy outcomes

Teacher support was consistently identified as one of the most influential contextual factors affecting reading literacy development among primary school pupil. Researcher generally agreed that teacher support encompassed affective support, instructional guidance, and evaluative feedback that helped students navigate academic challenges and maintain engagement in learning activities (Granziera et al., 2022; Prananto et al., 2025). Within literacy education, teacher support provided both academic resources and emotional reassurance that facilitated students’ participation in reading-related tasks. Numerous studies reported positive associations between teacher support and reading achievement, suggesting that supportive teacher-student relationships enhanced literacy outcomes across different educational setting (Guo et al., 2023). However, scholars differed regarding whether teacher support exerted a direct influence on reading literacy or whether its effects were transmitted through psychological mechanisms operating within learners. Some studies suggested that teacher support primarily functioned through students’ emotional experiences and motivational beliefs rather than through direct instructional effects alone (Liu et al., 2024). Consequently, teacher support was increasingly conceptualized as a multidimensional educational resource whose influence on reading literacy extended beyond classroom instruction.

Empirical evidence largely supported the positive relationship between teacher support and literacy outcomes. Studies consistently reported that students who perceived higher levels of teacher support demonstrated stronger reading engagement, greater persistence when confronted with challenging texts, and superior reading performance (Porter et al., 2024; Yang et al., 2021). Researchers further observed that supportive classroom environments encouraged students to devote greater cognitive effort to reading activities and to develop more positive attitudes toward literacy learning. These findings were particularly evident among younger learners, whose academic behaviors remained highly responsive to teacher feedback and social validation. Nevertheless, some investigations reported variations in the strength of the teacher support-literacy relationship across cultural contexts, age groups, and educational systems, indicating that contextual factors may moderate the observed effects. Furthermore, existing studies rarely examined the specific psychological processes through which teacher support translated into improved reading literacy. Therefore, although substantial evidence supported the educational importance of teacher support, important questions remained regarding the mechanisms underlying its influence on reading development.

Theoretical explanations of the teacher support-literacy relationship generally converged around social and motivational perspectives. Studies grounded in Self-Determination Theory suggested that teacher support fulfilled students’ needs for competence, autonomy, and relatedness, thereby fostering greater engagement and academic success (Ryan and Deci, 2023). Similarly, Social Cognitive Theory proposed that supportive teacher behaviors strengthened students’ confidence in their academic capabilities and encouraged persistence in challenging learning situations (Bandura, 2005). Empirical findings broadly supported both theoretical perspectives by demonstrating positive associations between teacher support, student engagement, and academic performance (Ray, 2020). However, these theories offered different explanations regarding the psychological pathways through which teacher support influenced literacy outcomes. While some researchers emphasized emotional processes, others focused on cognitive and motivational mechanisms. Consequently, further investigation was required to determine which psychological pathways most effectively explained the relationship between teacher support and reading literacy among primary school pupil.

#### 1.2.2 Emotional pathways linking teacher support and reading literacy

Achievement emotions emerged as an important psychological mechanism explaining students’ literacy development. According to Control-Value Theory, achievement emotions referred to emotional experiences directly associated with academic activities and learning outcomes, including both positive emotions such as enjoyment and pride and negative emotions such as anxiety, boredom, and hopelessness (Pekrun, 2024). Researchers generally agreed that achievement emotions influenced how students interpreted learning experiences and responded to academic challenges. Within literacy education, achievement emotions were found to shape students’ willingness to engage with reading materials, sustain concentration, and persist in difficult comprehension tasks. Numerous studies demonstrated that emotional experiences significantly predicted reading performance and academic engagement across different educational contexts (Hamedi et al., 2020). However, disagreement remained regarding which emotional dimensions exerted the strongest influence on literacy outcomes and whether positive and negative emotions operated symmetrically. Consequently, achievement emotions became a central focus in efforts to understand the psychological foundations of reading literacy development.

Research consistently demonstrated that positive achievement emotions promoted reading literacy outcomes. Studies reported that students who experienced enjoyment, confidence, and pride during literacy activities exhibited stronger reading comprehension, higher engagement, and greater willingness to participate in challenging academic tasks (Dewi, 2020). Positive emotional experiences appeared to broaden students’ cognitive resources and increase their persistence when encountering unfamiliar vocabulary or complex textual structures. These findings were particularly important among Grade 4 pupils because emotional self-regulation remained under development during middle childhood (Monteiro et al., 2021). Nevertheless, positive emotions alone could not fully explain variations in reading performance because some students continued to experience literacy difficulties despite reporting generally favorable emotional experiences. Moreover, positive emotional effects often varied according to classroom climate and individual learner characteristics. Therefore, although positive achievement emotions appeared beneficial for literacy development, their role required further examination within broader explanatory models.

Conversely, negative achievement emotions were frequently associated with poorer literacy outcomes. Studies consistently found that anxiety, boredom, and hopelessness were negatively correlated with reading comprehension, literacy engagement, and academic persistence (Hamedi et al., 2020). Researchers argued that these emotions consumed cognitive resources that would otherwise be available for reading comprehension and critical interpretation. High levels of anxiety often reduced students’ willingness to engage with challenging texts, whereas boredom undermined sustained attention and effort during literacy activities (Dewi, 2020). However, some scholars suggested that moderate levels of anxiety occasionally motivated greater preparation and effort, indicating that emotional influences may not always be uniformly negative. Furthermore, most existing studies examined emotional outcomes independently rather than as mechanisms linking social support and literacy achievement. Consequently, although achievement emotions clearly influenced reading literacy, their mediating role within the teacher support-literacy relationship remained insufficiently understood.

#### 1.2.3 Motivational pathways linking teacher support and reading literacy

Reading self-efficacy represented another important psychological mechanism associated with literacy development. Within Social Cognitive Theory, self-efficacy refers to the beliefs of individuals about their capability to successfully organize and execute actions required to achieve specific goals (Bandura, 2005). Within literacy contexts, reading self-efficacy referred to students’ confidence in their ability to complete reading tasks and achieve comprehension objectives (Bakkaloglu and Pilten, 2023). Researchers generally agreed that self-efficacy influenced students’ academic choices, effort expenditure, and persistence when confronted with challenging learning situations. Numerous studies reported positive associations between reading self-efficacy and literacy achievement across different educational settings. However, scholars continued to debate the relative influence of self-efficacy compared with other motivational and emotional variables. Consequently, reading self-efficacy remained a central construct in explanations of reading literacy development.

Empirical evidence generally supported the positive relationship between reading self-efficacy and literacy outcomes. Studies consistently found that students with stronger efficacy beliefs demonstrated higher levels of reading engagement, greater persistence, and superior reading comprehension performance (Bakkaloglu and Pilten, 2023; Yang et al., 2021). Researchers suggested that confident learners were more willing to attempt difficult reading tasks and were better able to employ effective literacy strategies when encountering obstacles. These findings aligned with evidence indicating that self-efficacy functioned as an important predictor of academic success across multiple learning domains (Ray, 2020). Nevertheless, some studies reported that high self-efficacy did not always translate into improved performance, particularly when students lacked adequate instructional support or effective reading strategies. Furthermore, efficacy beliefs were often influenced by contextual factors that extended beyond individual ability. Therefore, although reading self-efficacy appeared to be an important predictor of literacy outcomes, its effectiveness depended on broader educational conditions.

The development of reading self-efficacy was closely linked to social experiences within the classroom environment. Research indicated that teacher encouragement, constructive feedback, and supportive learning environments contributed significantly to the formation of positive efficacy beliefs among primary school pupils (Song, 2024). These influences were especially important during middle childhood because students continued to rely heavily on external evaluations when judging their own academic capabilities. Studies further demonstrated that supportive teacher-student relationships promoted greater confidence and willingness to engage in challenging literacy tasks. However, existing self-efficacy research often focused on individual cognitive processes while giving less attention to the social conditions that facilitated efficacy development. Moreover, few studies examined whether self-efficacy functioned as a mediating mechanism linking teacher support and reading literacy. Consequently, further investigation was required to clarify the motivational pathway through which teacher support influenced literacy outcomes.

#### 1.2.4 Unresolved mediation mechanisms and theoretical framework

Existing literacy provided substantial evidence that teacher support, achievement emotions, and reading self-efficacy were individually associated with reading literacy outcomes. Studies consistently demonstrated positive relationships between supportive classroom environments and literacy achievement, while separate bodies of research linked emotional experiences and efficacy beliefs to academic success (Pekrun, 2024; Bandura, 2005). Collectively, these findings suggested that teacher support influenced reading development through psychological processes operating within learners. Previous research therefore established a strong foundation for examining more complex explanatory mechanisms underlying literacy achievement. However, most studies investigated these relationships independently and rarely considered how emotional and motivational processes operated simultaneously. As a result, important theoretical questions remained regarding the relative contributions of affective and cognitive-motivational pathways to reading literacy development.

The unresolved nature of these mediation mechanisms was particularly evident among primary school pupils. Grade 4 learners occupied a critical developmental stage characterized by increasing academic demands, emerging self-regulatory capacities, and continued reliance on teacher guidance. Existing studies suggested that both achievement emotions and self-efficacy beliefs were highly sensitive to social experiences during this period (Song, 2024; Monteiro et al., 2021). Nevertheless, researchers rarely examined whether emotional experiences and efficacy beliefs functioned as complementary or competing mechanisms in explaining literacy outcomes. Furthermore, little empirical evidence existed regarding the relative strength of these pathways among Chinese primary school pupils. Consequently, the psychological processes through which teacher support influenced reading literacy during middle childhood remained insufficiently understood.

The need for further investigation was particularly relevant within the context of Confucian Heritage Culture. In Chinese educational settings, teachers traditionally occupied positions of considerable authority and influence, making teacher support a potentially powerful determinant of students’ emotional and motivational experiences (Chen, 2023). Previous studies suggested that teacher evaluations and feedback exerted stronger effects on students’ academic self-perceptions in CHC contexts than in many Western educational systems. However, existing theoretical models had rarely been tested using integrated frameworks that simultaneously considered emotional and motivational mechanisms within this sociocultural environment. As a result, uncertainly remained regarding whether achievement emotions or reading self-efficacy represented the more influential pathway through which teacher support affected literacy outcomes. Therefore, investigating these parallel mediation mechanisms within a Chinese context represented an important contribution to literacy and educational psychology research.

To address these gaps, the present study integrated Control-Value Theory and Social Cognitive Theory within a parallel mediation framework. Control-Value theory explained how teacher support could influence reading literacy through students’ achievement emotions, whereas Social Cognitive Theory explained how teacher support could shape literacy outcomes through reading self-efficacy beliefs. Together, these perspectives provided a comprehensive explanation of the affective and cognitive-motivational processes underlying reading development. Previous research had supported each pathway independently but had rarely examined them simultaneously among Chinese Grade 4 pupils. By investigating the parallel mediating roles of achievement emotions and reading self-efficacy, the present study sought to clarify the psychological mechanisms through which perceived teacher support promoted reading literacy. Consequently, this study proposed an integrated theoretical framework that addressed an important gap in existing literature and provided the basis for the hypotheses that followed.

*H1*: Students’ perceived teacher support has a significant and positive predictive effect on the reading literacy of Grade 4 pupils.

*H2*: Achievement emotions mediates the relationship between perceived teacher support and reading literacy.

*H3*: Reading self-efficacy mediates the relationship between perceived teacher support and reading literacy.

*H4*: Achievement emotions and reading self-efficacy function as parallel mediators.

## 2 Methodology

### 2.1 Research design and setting

This study adopted a quantitative cross-sectional survey design to examine the relationships among perceived teacher support, achievement emotions, reading self-efficacy, and reading literacy among Grade 4 pupils. A quantitative approach was considered appropriate because the study sought to test a theoretically derived parallel mediation model and estimate the magnitude of relationships among latent variables using statistical procedures. The study was grounded in a post-positivist paradigm, which assumes that psychological constructs can be measured objectively through validated instruments and analyzed using empirical methods. Previous educational studies investigating motivational and emotional mechanisms underlying academic achievement have successfully employed similar survey-based designs (Berndt, 2020). Although longitudinal designs provide stronger evidence regarding causal relationships, they often require substantial resources and extended access to school populations that may not be feasible in large-scale educational settings. Consequently, a cross-sectional survey design was considered an appropriate and efficient approach for examining the proposed theoretical framework among primary school pupils.

The study was conducted in two public primary schools located in Haidian District, Beijing, China. Haidian District was selected because it is widely recognized as one of the most academically competitive regions in China and maintains strong alignment with national curriculum standards and international literacy assessment frameworks such as PIRLS. The selected schools represented typical urban public schools operating within the context of Confucian Cultural Heritage, where traditional educational values coexisted with contemporary pedagogical practices. Previous research suggested that teacher-student interactions may exert particularly strong influences on students’ psychological development in such educational environments (Chen, 2023). Nevertheless, schools located in Haidian District may not fully represent rural or less-developed educational contexts in China. Therefore, the findings of this study should be interpreted primarily within the context of high-performing urban primary school.

### 2.2 Participants and sampling

The target population comprised Grade 4 pupils enrolled in public primary schools in Beijing, China. Grade 4 pupils were selected because this developmental stage represented a critical transition from basic decoding skills toward more advanced reading comprehension and literacy application abilities. During this period, students increasingly encountered complex reading materials and became more sensitive to classroom support, emotional experiences, and efficacy beliefs. Previous literacy research suggested that psychological factors played an increasingly important role in reading development during middle childhood. However, relatively few studies had examined these mechanisms simultaneously among Chinese Grade 4 pupils. Consequently, this population provided an appropriate context for investigating the psychological pathways linking teacher support and reading literacy.

A cluster sampling strategy was employed to recruit participants from the two participating schools. Cluster sampling was considered appropriate because it preserved the natural classroom structure, reduced administrative burden, and enhanced the feasibility of data collection in school settings (Berndt, 2020). All Grade 4 classes within the selected schools were invited to participate in the study. Prior to data collection, ethical approval was obtained from the relevant institutional review board, and informed consent was secured from school administrators, parents, and participating pupils. Previous educational studies have demonstrated that cluster sampling provides adequate representativeness while maintaining practical feasibility in school-based research. Although convenience and cluster sampling may limit generalizability compared with probability sampling methods, they remain widely accepted approaches in educational psychology research. Therefore, cluster sampling was considered suitable for the objectives and logistical constraints of the present study.

The inclusion criteria required participants to be enrolled as grade 4 pupils in one of the participating schools and to provide complete responses to all study instruments. Exclusion criteria included substantial missing data exceeding 20% of responses on any instrument, highly repetitive response patterns indicating inattentive completion, and duplicate questionnaire submissions. A total of 498 questionnaires were initially collected, and 38 responses were excluded following data screening procedures. The final sample consisted of 460 valid participants, which exceeded the minimum sample size recommendations for structural equation modeling. Methodological literature generally recommends sample sizes above 200 for SEM analyses, while larger samples contribute to greater parameter stability and statistical power. Consequently, the final sample size was considered sufficient for testing the proposed parallel mediation model.

In the results section, Table 1 provides a summary of the demographic characteristics of the participants, such as gender, age, the educational attainment of the father, the educational attainment of the mother, and whether they were an only child.

**Table 1:** Demographic profile of research participants.

Characteristics	Options	Frequency	Percentage (%)
Gender	Male	234	50.9
	Female	226	49.1
Age	9 years old	171	37.2
	10 years old	289	62.8
Household	Beijing household	345	75.0
	Non-Beijing resident	115	25.0
Father’s education	Junior high school	43	9.3
	High school	80	17.4
	Diploma	137	29.8
	Bachelor’s degree	155	33.7
	Postgraduate degree	45	9.8
Mother’s education	Junior high school	47	10.2
	High school	67	14.6
	Diploma	162	35.2
	Bachelor’s degree	144	31.3
	Postgraduate degree	40	8.7
Only child	Yes	127	27.6
	No	333	72.4
Total		460	100.0

### 2.3 Measures

This study employed four validated instruments to assess perceived teacher support, achievement emotions, reading self-efficacy and reading literacy. The selection of these instruments was guide by their theoretical alignment with the proposed conceptual framework and their established psychometric properties in educational research. Each instrument measured a distinct construct that was hypothesized to contribute to the development of reading literacy among Grade 4 pupils. Previous studies had demonstrated satisfactory reliability and validity for these instruments across different educational contexts (Bru et al., 2010; Pekrun et al., 2011; Koşar et al., 2022). Although, alternative instruments were available, the selected measures were considered most appropriate because they directly corresponded to the theoretical constructs examined in this study. Furthermore, the instruments had been widely used in literacy, motivation, and educational psychology research, facilitating comparison with previous findings. Consequently, the selected measures provided a robust foundation for evaluating the proposed parallel mediation model.

#### 2.3.1 Translation and pilot testing

All instruments were administered in Chinese to ensure that participants fully understood the questionnaire items and response options. Where validated Chinese versions were unavailable, a rigorous translation and back-translation procedure was employed to establish linguistic and conceptual equivalence. Two bilingual researchers independently translated the original English instruments into Chinese, after which a third bilingual scholar performed a back-translated into English. Discrepancies between the original and back-translation versions were reviewed and resolved by an expert panel consisting of educational researchers and literacy specialists. Although minor linguistic adaptations were required to improve age appropriateness for Grade 4 pupils, care was taken to preserve the conceptual meaning of each item. Consequently, the final Chinese versions were considered linguistically and culturally suitable for the target population.

A pilot study was conducted prior to formal data collection to evaluate instrument clarity and psychometric adequacy. Fifty Grade 4 pupils from a public primary school in Haidian District, Beijing, participated in the pilot study. Participants completed all instruments under conditions similar to those used in the main study, allowing the research team to evaluate comprehension, response patterns, and completion time. Previous methodological literature has emphasized the importance of pilot testing in identifying potential measurement problems before large-scale administration. Although pilot samples are typically smaller than the final study sample, they provide valuable preliminary evidence regarding instrument suitability. Therefore, the pilot study served as an important quality assurance procedure before formal data collection commenced.

The pilot study results indicated satisfactory psychometric performance across all instruments. Cronbach’s alpha coefficients ranged from 0.94 to 0.95, exceeding the recommended threshold of 0.70. These findings suggested that the instruments possessed adequate internal consistency. Although psychometric properties may vary slightly in large samples, the pilot study results provided strong preliminary support for the reliability of the measurement instruments. Consequently, all instruments were retained for use in the main study without substantial modification.

#### 2.3.2 Perceived teacher support

Perceptions of teacher support were measured using the Student Perceived Teacher Support Questionnaire, which has been adapted for the specific academic context of primary school pupils (Bru et al., 2010). This instrument is designed to assess the extent to which pupils perceive that teachers provide support in promoting their emotional well-being and academic development. The questionnaire comprises 16 items, divided into four dimensions: Teacher’s Academic Support (4 items; e.g., “When the students do group work, teachers explain well.”) Teacher’s Emotional Support (5 items; e.g., “I feel the teachers care about the me.”) Teacher’s Autonomy Support (3 items; e.g., “I participate in decisions regarding choice of my learning tasks.”) and Teacher’s Managerial Support (4 items; e.g., “The teachers make sure we do our best in class.”) Participants rated each item using a 4-point Likert scale, ranging from SD (strongly disagree) to SA (strongly agree), with higher scores indicating a greater degree of teacher support perceived by the student. This multidimensional design allows for a detailed assessment of how different types of teacher-student interactions influence students’ psychological resources. In this study, the scale demonstrated excellent internal consistency, with highly robust reliability coefficients for each subscale ranging from 0.69 to 0.86, confirming that the scale is a highly reliable tool for assessing social support in the context of Chinese basic education.

#### 2.3.3 Achievement emotions

To assess students’ emotional experiences during literacy tasks, this study employed the Achievement Emotions Questionnaire (AEQ) (Pekrun et al., 2011). This scale has been specifically adapted to measure specific achievement emotions most relevant to primary school pupils’ academic performance. The questionnaire covers both positive and negative emotional states, comprehensively reflecting students’ psychological responses during reading activities (Dewi, 2020). The revised version of the questionnaire used in this study comprises 24 items, divided into three core dimensions: Classroom Achievement Emotions (8 items, e.g., ‘I enjoy being in this class’), Learning Achievement Emotions (8 items, e.g., ‘I enjoy acquiring new knowledge’) and Test Achievement Emotions (8 items, e.g., ‘For me, the test was challenging and enjoyable’). All responses were rated on a 5-point Likert scale ranging from SD (strongly disagree) to SA (strongly agree), with negative emotion items reverse-scored to ensure consistency in data interpretation. This multidimensional research approach aligns with Control Value Theory (CVT) and enables a comprehensive analysis of emotional experiences. This study examined how social support influences various emotional responses (Pekrun, 2024). Research indicated that the AEQ scale exhibits high internal consistency, Cronbach’s alpha values for both would be above 0.70, respectively suggesting that the questionnaire is a reliable and valid tool for measuring the emotional mechanisms underlying reading literacy.

#### 2.3.4 Reading self-efficacy

Reading self-efficacy was assessed using the Reading Self-Efficacy Scale, which has been specifically adapted to evaluate students’ internal beliefs regarding their literacy to complete various reading tasks. Designed on the basis of social cognitive theory, the scale reflects students’ levels of self-efficacy in organizing and executing the actions required to achieve specific reading comprehension goals (Koşar et al., 2022). The scale used in this study comprises 15 items, with Reading self-efficacy (e.g., ‘I can identify the topic of a reading passage.’) Participants responded using a 5-point Likert scale, with scores ranging from SA (strongly disagree) to SD (strongly agree). This measurement method aligns with the developmental characteristics of Grade 4 pupils, who increasingly rely on external feedback to form judgment about their own abilities. In this study, the scale demonstrated high internal consistency, with a Cronbach’s alpha coefficient of 0.9, indicating that the scale possesses satisfactory convergent validity and reliability in measuring cognitive motivational mechanisms within the context of Chinese basic education.

#### 2.3.5 Reading literacy

Reading literacy was assessed using the Chinese adaptation of the Progress in International Reading Literacy Study (PIRLS) 2016 reading assessment. PIRLS was selected because it is one of the most widely recognized international frameworks for evaluating primary school students’ reading literacy and has been extensively applied in comparative educational research. The assessment measured students’ ability to extract information, make inferences, interpret and integrate textual information, and evaluate written content. Previous studies have demonstrated that PIRLS provides a comprehensive assessment of literacy skills that extend beyond simple decoding and vocabulary recognition. Although other reading assessments are available, PIRLS was considered particularly suitable because its conceptualization of reading literacy aligns closely with contemporary literacy education frameworks. Consequently, the PIRLS assessment provided a valid measure of students’ reading literacy performance.

The reading assessment utilized the Chinese version of the PIRLS literary text “Enemy Pie,” which consisted of 16 items combining multiple-choice and constructed-response formats. Students reading literacy scores were calculated using the original PIRLS scoring procedures. Multiple-choice items were scored as either correct or incorrect, whereas constructed-response items were scored according to standardized scoring rubrics provided within the PIRLS framework. The total reading literacy score ranged from 0 to 20, with higher scores indicating stronger reading literacy performance. Previous methodological literature has recommended retaining continuous achievement scores whenever possible because score categorization reduces variance and statistical precision. Although categorical classifications may simplify interpretation, they can substantially reduce statistical power and obscure meaningful differences among participants. Consequently, raw reading literacy scores were retained and analyzed as continuous variables throughout all statistical procedures.

### 2.4 Procedure

The data collection process strictly adhered to ethical research guidelines and was conducted in accordance with the ethical principles outlined in the Declaration of Helsinki. Ethical approval was obtained from the institutional Ethics Committee of Northeastern University, China (Approval No.: NJCIT/IEC-2023-015) prior to data collection, and permission was subsequently obtained from the participating schools in Beijing.

To resolve the methodological challenge of linking parental demographic information with students’ assessment data while strictly preserving participant anonymity, data collection was conducted via a unified online survey platform accessed through a secure link. Under the guidance of legal guardians, students completed the entire evaluation on their parents’ devices at home or in a designated online setting during the mid-term of the academic term. This integrated procedure allowed parental consent and demographic information to be captured directly alongside the students’ responses to the PIRLS-based reading literacy assessment, the Achievement Emotions Questionnaire, the Reading Self-Efficacy Scale, and the Perceived Teacher Support Questionnaire.

To safeguard privacy and ensure complete confidentiality, no personally identifiable information (such as real names or student ID numbers) was requested or recorded by the system; instead, anonymous system-generated tokens or non-identifiable identifiers were used internally by the platform to link the household demographic variables with the student’s assessment scores within a password-protected database. Data that did not meet quality standards (e.g., incomplete submissions or duplicate entries) were excluded from the final analysis. Participation was entirely voluntary, and all participants and their legal guardians were informed of their right to withdraw from the study at any stage without penalty.

### 2.5 Data analysis

Data analysis was conducted in four sequential stages comprising descriptive statistics, common method bias assessment, measurement model evaluation, and structural model testing. This analytical strategy was designed to ensure that the quality of the data and measurement instruments was established before testing the hypothesized relationships among latent variables. Previous structural equation modeling research has recommended a systematic analytical approach that evaluates both measurement quality and structural relationships (Hair et al., 2021). Although individual analytical techniques provide valuable information, their combined application strengthens the credibility and robustness of empirical findings. Therefore, a multi-stage analytical framework was adopted to provide a comprehensive evaluation of the proposed theoretical model.

Descriptive statistics were first computed using IBM SPSS Statistics Version 26. Modes, medians were examined to evaluate data distribution and identify potential violations of statistical assumptions. Pearson correlation coefficients were subsequently calculated to examine preliminary relationships among perceived teacher support, achievement emotions, reading self-efficacy, and reading literacy. Previous quantitative studies have routinely employed these procedures to establish the suitability of data for multivariate analyses. Although descriptive statistics alone cannot establish causal relationships, they provide important insights into the characteristics of the sample and study variables. Consequently, descriptive analyses served as the foundation for subsequent measurement and structural model evaluations.

Common method bias was assessed prior to hypothesis testing because several study variables were measured using self-report instruments. Harman’s single-factor test was performed by entering all self-report measurement items into an unrotated exploratory factor analysis. The first unrotated factor accounted for 33.4% of the total variance, which was below the commonly recommended threshold of 50%. Previous methodological research has suggested that substantial common method bias is unlikely when a single factor does not explain the majority of variance within the data. Although Harman’s single-factor test is not a definitive diagnostic procedure, it remains one of the most widely reported methods in educational and psychological research. Therefore, the results suggested that common method bias was unlikely to pose a serious threat to the validity of the findings in the present study.

Measurement model evaluations was conducted using AMOS Version 24. Confirmatory factor analysis (CFA) was performed to assess the reliability and validity of the latent constructs. Standardized factor loadings, composite reliability (CR), and average variance extracted (AVE) were examined to evaluate convergent validity, whereas discriminant validity was assessed by comparing the square root of AVE values with inter-construct correlations. Previous SEM literature has consistently recommended these procedures as indicators of satisfactory measurement quality. Although no single fit index is sufficient to evaluate model adequacy, the combined examination of multiple indices provides a more comprehensive assessment. Therefore, measurement model quality was evaluated using a combination of reliability, validity, and model-fit indicators.

Structural model testing was subsequently performed to examine the hypothesized direct and indirect relationships among study variables. Standardized path coefficients (β) and coefficients of determinations (R^2^) were calculated to evaluate the explanatory power of the model. The significance of direct and mediation effects was assessed using bias-corrected bootstrapping procedures with 5,000 resamples. Precious mediation research has recommended bootstrapping because it provides more accurate estimates of indirect effects than traditional significance testing methods. Although alternative mediation procedures are available, bootstrapping has become the preferred approach in contemporary SEM research. Consequently, bootstrapping was employed to provide robust estimates of the direct, indirect, and total effects specified within the proposed parallel mediation model.

## 3 Results

### 3.1 Sample characteristics

A total of 500 questionnaires were distributed to Grade 4 pupils in Beijing. Following rigorous data screening (including the removal of incomplete responses and the identification of extreme response patterns such as ‘linear’ or ‘extremely monotonous’ patterns), 460 valid questionnaires were ultimately retained for analysis. The response rate for valid questionnaires was 92%, providing a reliable statistical basis for assessing the structural relationships within the proposed parallel mediation model.

The demographic characteristics of the participants are summarized in Table 1. Of the 460 valid respondents, 234 were boys (50.9%) and 226 were girls (49.1%), indicating a balanced gender distribution within the group. All participants were full-time Grade 4 pupils, with an average age of 9–10 years. This age group is at a critical stage of cognitive and psychological development, with their learning and comprehension abilities gradually evolving toward greater independence and autonomy. The homogeneity of the sample in terms of year group and educational environment ensures that the findings regarding perceptions of teacher support, achievement emotions, reading self-efficacy and reading literacy accurately reflect the characteristics of this specific developmental stage within the context of urban education in China.

### 3.2 Descriptive statistics and correlation analysis

#### 3.2.1 Descriptive statistics and normality

To examine the data distribution for the study variables, descriptive statistics were evaluated using the frequency distributions, medians, and modes presented in Tables 2, 3. The central tendencies of the responses indicate that the participating Grade 4 pupils generally perceive a supportive teaching environment and possess positive psychological resources.

**Table 2:** Descriptive statistics of perceived teacher support.

Variables	Attitude				Median	Mode
	SD	D	A	SA		
TES1	35	111	122	192	A	SA
TES2	29	121	108	202	A	SA
TES3	28	125	99	208	A	SA
TES4	26	131	89	214	A	SA
TES5	29	123	91	217	A	SA
TAuS1	33	127	109	191	A	SA
TAuS2	37	125	117	181	A	SA
TAuS3	31	124	110	195	A	SA
TMS1	36	114	109	201	A	SA
TMS2	34	111	100	215	A	SA
TMS3	39	123	91	207	A	SA
TMS4	30	120	102	208	A	SA
TAcS1	32	121	89	218	A	SA
TAcS2	23	134	83	220	A	SA
TAcS3	39	125	76	220	A	SA
TAcS4	28	128	77	227	A	SA

PTS, perceived teacher support; TES, teachers’ emotional support; TAuS, teachers’ autonomy support; TMS, teachers’ managerial support; TAcS, teachers’ academic support; SD, strongly disagree; D, disagree; A, agree; SA, strongly agree.

**Table 3:** Descriptive statistics of achievement emotions and reading self-efficacy.

Variables	Attitude					Mode	Median
	SD	D	N	A	SA		
CAE1	23	114	83	127	113	A	A
CAE2	21	104	103	119	113	A	A
CAE3	28	94	105	116	117	A	SA
CAE4	15	103	108	125	109	A	A
CAE5	27	111	85	125	112	A	A
CAE6	27	99	98	130	106	A	A
CAE7	35	89	104	123	109	A	A
CAE8	23	111	97	118	111	N	A
LAE1	28	96	84	147	105	A	A
LAE2	22	96	95	138	109	A	A
LAE3	21	105	99	130	105	A	A
LAE4	24	87	125	124	100	N	N
LAE5	28	96	90	145	101	A	A
LAE6	24	93	99	133	111	A	A
LAE7	12	104	103	131	110	A	A
LAE8	20	96	102	148	94	A	A
TAE1	26	114	100	112	108	N	D
TAE2	23	105	99	123	110	A	A
TAE3	25	96	96	123	120	A	A
TAE4	24	104	112	109	111	N	N
TAE5	26	98	97	128	111	A	A
TAE6	25	89	114	133	99	A	A
TAE7	20	98	107	131	104	A	A
TAE8	36	79	91	130	124	A	A
RSE1	19	123	99	138	81	N	A
RSE2	20	110	114	141	75	N	A
RSE3	13	126	114	125	82	N	D
RSE4	17	113	105	146	79	N	A
RSE5	13	119	114	136	78	N	A
RSE6	14	130	109	122	85	N	D
RSE7	11	106	117	150	76	N	A
RSE8	17	113	104	156	70	N	A
RSE9	22	111	98	159	70	N	A
RSE10	19	114	94	154	79	A	A
RSE11	18	112	103	156	71	N	A
RSE12	22	107	113	150	68	N	A
RSE13	18	113	96	142	91	A	A
RSE14	19	98	119	151	73	N	A
RSE15	20	122	99	141	78	N	A

AE, achievement emotions; CAE, classroom achievement emotions; LAE, learning achievement emotions; TAE, test achievement emotions; RSE, reading self-efficacy; SD, strongly disagree; D, disagree; N, neutral; A, agree; SA, strongly agree.

Table 2 illustrates the descriptive statistics for perceived teacher support. Across all items measuring emotional, autonomy, managerial, and academic support, the median response was consistently agree. Furthermore, the mode for every single item in this category was strongly agree. This concentration of responses at the highest end of the measurement scale demonstrates a robust and highly positive perception of teacher support among the students.

Table 3 details the response distributions for achievement emotions and reading self-efficacy. For the items measuring achievement emotions across classroom, learning, and test settings, the median responses were predominantly agree, with only a few items recording a median of neutral. The most frequent responses for these emotional items were also primarily agree, alongside a few neutral selections. This pattern reflects generally positive emotional engagement during reading tasks.

Regarding reading self-efficacy, the results in Table 3 show a slightly different distribution. The median responses remained primarily at agree, although two specific items recorded a median of disagree. Notably, the mode for the vast majority of the reading self-efficacy items was neutral. This distribution suggests that while the overall central tendency leans positive, a significant portion of the students opted for a neutral stance when explicitly assessing their own reading capabilities.

#### 3.2.2 Correlations between key variables

To explore preliminary associations between latent constructs, this study calculated Pearson’s correlation coefficients between perceived teacher support, achievement emotions, reading self-efficacy and reading literacy. As shown in Table 4, all core variables exhibited significant positive correlations, which is consistent with the direction of the assumptions of the theoretical model. Specifically, perceived teacher support was moderately to highly positively correlated with reading literacy (*r* = 0.54, *p* < 0.01), indicating that students who perceive a higher level of teacher support tend to demonstrate superior literacy skills. Furthermore, perceived teacher support was positively correlated with both mediating variables: achievement emotions and reading self-efficacy. This pattern suggests that a supportive teaching environment is closely linked to the cultivation of students’ positive emotional states and levels of intrinsic confidence. Similarly, both achievement emotions and reading self-efficacy were significantly positively correlated with reading literacy, with correlation coefficients of *r* = 0.38 (*p* < 0.01) and *r* = 0.31 (*p* < 0.01), respectively. This provides preliminary empirical support for the proposed parallel mediation pathways. Overall, the correlations among the four main variables were moderate, indicating meaningful structural relationships while providing sufficient conceptual distinction for subsequent structural equation modeling analysis.

**Table 4:** Pearson correlation matrix of main variables.

Variable	1	2	3	4
1. PTS	1	–	–	–
2. AE	0.503***	1	–	–
3. RSE	0.392***	0.417***	1	–
4. READ_LIT	0.540***	0.383***	0.308***	1

PTS, perceived teacher support; AE, achievement emotions; RSE, reading self-efficacy; READ_LIT, reading literacy; ***p < 0.001, **p < 0.01, *p < 0.05.

### 3.3 Measurement model

#### 3.3.1 Construct validity and reliability

This study confirmed that the scales possessed good construct validity through exploratory factor analysis (EFA), as shown in Table 5. Based on KMO values of ≥0.73 for all scales and a *p*-value of <0.001 for Bartlett’s sphericity test, confirmatory factor analysis (CFA) was further employed to evaluate the measurement model, ensuring the reliability and convergent validity (aggregate validity) of the latent constructs. As shown in Table 6, the standardized factor loadings for the first-order factors of perceived teacher support, achievement emotions and reading self-efficacy ranged from 0.706 to 0.853, all exceeding the recommended threshold of 0.50, indicating that the observed indicators effectively reflect their respective latent variables. Convergent validity and reliability were further tested using Average Variance Extracted (AVE) and Composite Reliability (CR): the AVE values for all first-order factors ranged from 0.568 to 0.682, while the CR values ranged from 0.850 to 0.952. Furthermore, the results of the second-order measurement model (see Table 7) indicate that the AVE values for the three core constructs which perceived teacher support, achievement emotions, and reading self-efficacy (ranging from 0.568 to 0.602) all exceeded 0.50, while the CR values (ranging from 0.820 to 0.952) all met the standard requirement of ≥0.70. Furthermore, all values satisfied commonly recommended criteria, indicating that the measurement model adequately represented the observed data (see Table 8). Furthermore, Cronbach’s alpha coefficients (0.94 to 0.95) were observed (see Table 7), with all scales and sub-dimensions achieving Cronbach’s alpha values of ≥0.85, significantly exceeding the standard threshold of 0.80 for good reliability. These results indicate that the constructs effectively explain the majority of the variance in the measurement indicators, confirming that the scales possess excellent internal consistency. Taken together, the above indicators provide strong evidence that the measurement tools used in this study are highly reliable and possess sufficient convergent validity for subsequent structural equation modeling tests.

**Table 5:** KMO and Bartlett’s sphericity test results for each scale.

Scale	Number of items	KMO	Bartlett’s *χ*^2^	df	*p*
TES	5	0.891	1383.19	10	<0.001
TAuS	3	0.732	588.02	3	<0.001
TMS	4	0.839	1073.45	6	<0.001
TAcS	4	0.844	1046.18	6	<0.001
PTS	16	0.942	4919.62	120	<0.001
CAE	8	0.946	2431.91	28	<0.001
LAE	8	0.945	2203.06	28	<0.001
TAE	8	0.945	2308.19	28	<0.001
AE	24	0.967	7601.71	276	<0.001
RSE	15	0.980	4417.72	105	<0.001

PTS, perceived teacher support; TES, teachers’ emotional support; TAuS, teachers’ autonomy support; TMS, teachers’ managerial support; TAcS, teachers’ academic support; AE, achievement emotions; CAE, classroom achievement emotions; LAE, learning achievement emotions; TAE, test achievement emotions; RSE, reading self-efficacy; KMO, Kaiser–Meyer–Olkin; df, degrees of freedom.

**Table 6:** Confirmatory factor analysis: first-order factor convergent validity.

First-order factor	Number of items	Range of standardized loadings	AVE	CR
TES	5	0.790–0.833	0.658	0.906
TAuS	3	0.807–0.811	0.654	0.850
TMS	4	0.789–0.843	0.682	0.896
TAcS	4	0.797–0.853	0.677	0.893
CAE	8	0.758–0.829	0.626	0.930
LAE	8	0.763–0.789	0.599	0.923
TAE	8	0.756–0.800	0.611	0.926
RSE	15	0.706–0.793	0.568	0.952

AVE, average variance extracted; CR, composite reliability.

**Table 7:** Confirmatory factor analysis: second-order convergent validity.

Concept	Cronbach’s alpha	AVE	CR	√AVE
PTS	0.939	0.588	0.851	0.767
AE	0.954	0.602	0.820	0.776
RSE	0.952	0.568	0.952	0.753

PTS, perceived teacher support; AE, achievement emotions; RSE, reading self-efficacy; AVE, average variance extracted; CR, composite reliability.

**Table 8:** Second-order structural equation model fit indices.

Indicator	Estimated value	Standard	Assessment
*χ* ^2^	2095.87	–	–
df	1,474	–	–
*χ*^2^/df	1.422	<3 Good	Excellent
CFI	0.964	>0.95 Excellent	Excellent
TLI	0.963	>0.95 Excellent	Excellent
GFI	1.000	>0.95 Excellent	Excellent
RMSEA	0.030	<0.06 Excellent	Excellent

*x*^2^, likelihood-ratio; df, degrees of freedom; *x*^2^/df, Chi-square test; CFI, comparative fit index; TLI, Tucker–Lewis index; GFI, goodness fit index; RMSEA, root mean square error of approximation.

#### 3.3.2 Discriminant validity

To ensure that each latent construct in the model is empirically independent of the others, we assessed their discriminant validity. According to the Fornell–Larcker criterion, a construct is deemed to possess discriminant validity when the square root of its average variance extracted (AVE) is greater than its correlation coefficient with other constructs in the model. As shown in Table 9, the square roots of the AVE for Perceived Teacher Support (0.767), Achievement Affect (0.776) and Reading Self-Efficacy (0.753) were all significantly higher than the off-diagonal correlation coefficients between these variables (ranging from 0.392 to 0.503). For example, the highest observed correlation coefficient occurred between perceived teacher support and achievement emotions (*r* = 0.503), a value significantly lower than the respective square roots of the AVEs for these two constructs (0.767 and 0.776). These results indicate that the three latent variables in the model possess good discriminant validity, confirming that they represent distinct psychological and academic constructs in the structural model and are suitable for inclusion in the same structural equation model for subsequent analysis.

**Table 9:** Discriminant validity test (Fornell–Larcker criteria).

Concept	PTS	AE	RSE
PTS	**0.767**	0.503	0.392
AE	0.503	**0.776**	0.417
RSE	0.392	0.417	**0.753**

The bolded values on the diagonal represent the √AVE for each construct; the values below the diagonal represent the inter-correlation coefficients between constructs.

### 3.4 Common method bias

Harman’s single-factor test was conducted to assess the potential influence of common method bias. The first unrotated factor accounted for 33.4% of the total variance (see Table 10), which was below the recommended threshold of 50%. Therefore, common method bias was unlikely to represent a substantial threat to the validity of the findings.

**Table 10:** Harman’s single-factor test for common method bias.

Factor number	Initial eigenvalue	Percentage of variance explained (%)	Cumulative percentage of variance explained (%)
1	18.366	33.392	33.392
2	5.894	10.717	44.109
3	4.757	8.649	52.758
4	2.274	4.135	56.894
5	2.086	3.793	60.687
6	1.466	2.666	63.352
7	1.253	2.279	65.631
8	1.060	1.928	67.559

Only the eight factors with eigenvalues greater than 1 are listed.

### 3.5 Structural model

#### 3.5.1 *R*^2^ and explanatory power

After establishing an appropriate measurement model, the explanatory power of the structural model was tested. As shown in Figure 1 and Table 11, perceived teacher support (PTS) and the two mediating variables jointly explained the following proportions of variance in the endogenous variables: the model explained 25.3% of the variance in achievement emotions (AE; *R*^2^ = 0.253, adjusted *R*^2^ = 0.251), reading self-efficacy (RES; *R*^2^ = 0.154, adjusted *R*^2^ = 0.152) 15.4% of the variance, and reading literacy (READ_LIT; *R*^2^ = 0.313, adjusted *R*^2^ = 0.309) 31.3% of the variance. Overall, the *R*^2^ results indicate that the explanatory power of the model for reading literacy is moderate, while its explanatory power for achievement emotions and reading self-efficacy is weak to moderate. These figures indicate that perceived teacher support and these two mediating variables account for a significant proportion of the variance in a reading comprehension teaching context. Specifically, the structural paths from perceived teacher support to these two mediating variables, and from perceived teacher support and the mediating variables to reading literacy, account for some of the variance in students’ psychological functioning and objective performance during reading tasks; however, they also suggest that there remains considerable scope for the influence of other unmeasured determinants.

**Figure 1:**
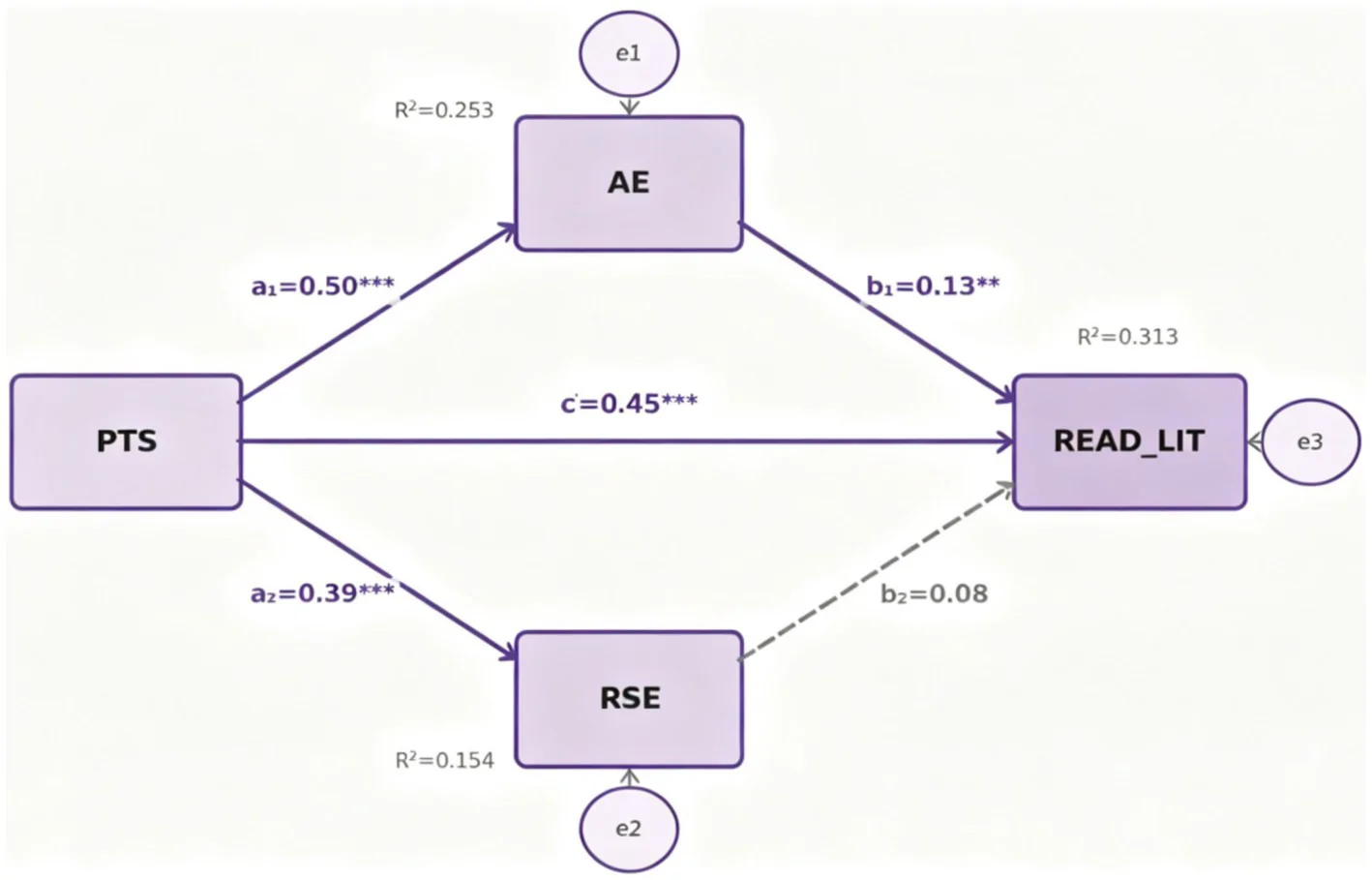
Structural equation model path diagram (standardized estimates). ***Indicates high statistical significance at *p* < 0.001 (less than a 0.1% probability that the observed relationship occurred by chance). **Indicates statistical significance at *p* < 0.01 (less than a 1% probability that the observed relationship occurred by chance).

**Table 11:** Explanatory power of endogenous variables.

Endogenous variables	*R* ^2^	Adjusted *R*^2^
AE	0.253	0.251
RSE	0.154	0.152
READ_LIT	0.313	0.309

AE, achievement emotions; RSE, reading self-efficacy; READ_LIT, reading literacy.

#### 3.5.2 Path coefficients

The results of the structural path estimates are summarized in Table 12 and illustrated in the corresponding figure. Perceived teacher support (PTS) has a significant positive direct effect on achievement emotions (*β* = 0.503, *p* < 0.001) and reading self-efficacy (*β* = 0.392, *p* < 0.001). Furthermore, the direct path from PTS to reading literacy (READ_LIT) was found to be significantly positive (*β* = 0.446, *p* < 0.001), supporting Hypothesis H1. These results indicate that students who perceive greater teacher support in reading courses tend to report more positive achievement emotions and higher self-efficacy, and their objective reading performance is also superior.

**Table 12:** Estimated path coefficients for the structural model.

Path	Variable relationship	Estimate	S. E.	C. R.	*P*	Standardized *β*	Significance
a₁	PTS → AE	0.590	0.047	12.457	<0.001	0.503	***
a₂	PTS → RSE	0.477	0.052	9.129	<0.001	0.392	***
c′	PTS → READ_LIT	0.619	0.066	9.330	<0.001	0.446	***
b₁	AE → READ_LIT	0.149	0.053	2.813	0.005	0.126	**
b₂	RSE → READ_LIT	0.093	0.048	1.929	0.054	0.081	ns

PTS, perceived teacher support; AE, achievement emotions; RSE, reading self-efficacy; READ_LIT, reading literacy; Estimate, unstandardised estimate; C. R., critical ratio (Estimate/S. E.); ***p < 0.001, **p < 0.01, ns not significant.

Furthermore, regarding the relationship between the two mediating variables and reading literacy (READ_LIT): achievement emotions exhibited a significant positive path relationship with reading literacy (*β* = 0.126, *p* = 0.005). However, the direct path from reading self-efficacy to reading literacy was not statistically significant, reaching only marginal significance (*β* = 0.081, *p* = 0.054). Taken together, these results indicate that perceived teacher support not only directly enhances students’ objective reading literacy (supporting H1) but also exerts a positive indirect facilitating effect primarily via achievement emotions (supporting H2), whereas the independent direct path through reading self-efficacy did not reach statistical significance in this specific model (failing to independently support H3). As will be confirmed in the subsequent mediation analyses, the overall parallel mediation framework remains supported (H4).

#### 3.5.3 Effect sizes and predictive correlations

Effect sizes for the structural path were assessed using Cohen’s *f*^2^. The results are shown in Table 13. Perceived teacher support (PTS), as the most critical predictor variable in the model, exhibited moderate-sized effects on all endogenous variables: it had a moderate effect on reading literacy (READ_LIT) (*f*^2^ = 0.204), a moderate effect on reading self-efficacy (RSE) (*f*^2^ = 0.182), and a moderate effect on achievement emotions (AE) (*f*^2^ = 0.338). By contrast, the independent pathways of achievement emotions and reading self-efficacy to reading literacy exhibited only extremely weak effect sizes (*f*^2^ = 0.016 and *f*^2^ = 0.007, respectively), suggesting that the marginal contribution of these two psychological mediating variables to students’ objective reading literacy is relatively limited once perceived teacher support is controlled for. In terms of the overall explanatory power of the model, the results showed that the model explained 25.3% of the variance in achievement emotions (*R*^2^ = 0.253, adjusted *R*^2^ = 0.251), 15.4% of the variance in reading self-efficacy (*R*^2^ = 0.154, adjusted *R*^2^ = 0.152), and 31.3% of the variance in reading literacy (*R*^2^ = 0.313, adjusted *R*^2^ = 0.309). The explanatory power of these three endogenous variables falls within the acceptable and reasonable range for social science research. Overall, the model provides valuable insights into reading teaching contexts, indicating that perceived teacher support and the emotional experiences it elicits can account for a significant portion of the variation in students’ reading performance.

**Table 13:** Cohen’s *f*^2^ effect sizes for each structural path.

Path	Variable relationship	*f* ^2^	Effect size
a₁	PTS → AE	0.338	Moderate effect
a₂	PTS → RSE	0.182	Moderate effect
c′	PTS → READ_LIT	0.204	Moderate effect
b₁	AE → READ_LIT	0.016	Weak effect
b₂	RSE → READ_LIT	0.007	Small effect

PTS, perceived teacher support; AE, achievement emotions; RSE, reading self-efficacy; READ_LIT, reading literacy; *f*^2^ ≥ 0.02 indicates a small effect, ≥0.15 indicates a moderate effect, and ≥0.35 indicates a large effect.

### 3.6 Mediation analysis

This study employed a bias-corrected non-parametric bootstrap method (with 5,000 repetitions) to test for mediating effects. A mediating effect is considered significant when the 95% confidence interval does not include zero. The analysis examined whether achievement emotions (AE) and reading self-efficacy (RSE) mediated the relationship between perceived teacher support (PTS) and reading literacy (READ_LIT).

As shown in the relevant results (see Table 14), the indirect effect of perceived teacher support (PTS) on reading literacy (READ_LIT) via achievement emotions (AE) is statistically significant (PTS → AE → READ_LIT: *β* = +0.063, 95% CI = [+0.015, +0.115], excluding zero). This suggests that higher perceived teacher support is not only associated with more positive emotions in reading contexts but also predicts higher levels of objective reading achievement. This result supports the hypothesis that achievement emotions (AE) acts as a mediator (H2). However, the indirect effect of PTS on READ_LIT via reading self-efficacy (RSE) was not statistically significant (PTS → RSE → READ_LIT: *β* = +0.032, 95% CI = [−0.002, +0.066]), as the confidence interval crossed the zero value. This suggests that, although reading self-efficacy is significantly positively correlated with both perceived teacher support and reading literacy, after controlling for achievement emotions, students who perceived stronger teacher support did not effectively establish an independent mediating pathway driving a significant improvement in objective scores, thereby failing to confirm the independent mediating role of RSE (H3). Importantly, after including both mediators in the model, the total parallel mediating effect of achievement emotions and reading self-efficacy combined reached statistical significance (*β* = +0.095, 95% CI = [+0.044, +0.148], supporting H4);and the direct path from PTS to READ_LIT remained highly statistically significant (*β* = +0.541, *p* < 0.001, supporting H1). Taken together, this model constitutes a partial mediation model. These findings support the core tenets of the proposed model: perceived teacher support not only directly promotes students’ reading literacy but also indirectly enhances reading performance by stimulating students’ achievement emotions. This suggests that, among Grade 4 pupils, emotional mechanisms may play a more dominant role than self-efficacy beliefs in the process by which teacher support is translated into reading literacy. From a practical perspective, a more supportive environment in reading lessons can not only effectively improve pupils’ emotional experiences but also tangibly enhance their ultimate reading performance; however, educators may need to combine this with more targeted cognitive strategies in the future to help pupils transform perceived support into more robust reading self-efficacy.

**Table 14:** Results of bootstrap mediation analysis.

Effect	Path	Standardized *β*	95% CI	Proportion of total effect	Conclusion
Ind₁	PTS → AE → READ_LIT	+0.063	[+0.015, +0.115]	11.6%	Significant★
Ind₂	PTS → RSE → READ_LIT	+0.032	[−0.002, +0.066]	5.9%	Not significant
Total indirect effect	Σ Indirect	+0.095	[+0.044, +0.148]	17.6%	Significant★
Direct effect	PTS → READ_LIT (c′)	+0.446	–	82.4%	Significant★
Total effect	PTS → READ_LIT (c)	+0.541	–	100.0%	Significant★

PTS, perceived teacher support; AE, achievement emotions; RSE, reading self-efficacy; READ_LIT, reading literacy; Ind₁, the mediating effect of achievement emotions; Ind₂, the mediating effect of reading self-efficacy.

## 4 Discussion

This study was examined the relationships among perceived teacher support, achievement emotions, reading self-efficacy, and reading literacy among Grade 4 pupils in Beijing. Drawing upon self-determination theory, control-value theory and social cognitive theory, the study proposed that teacher support would influence reading literacy through both emotional and cognitive-motivation pathways. The results generally supported the proposed framework, although important differences emerged between the two mediating mechanism. Specifically, achievement emotions significantly mediated the relationship between perceived teacher support and reading literacy, whereas reading self-efficacy did not demonstrate an independent mediating effect. Furthermore, perceived teacher support maintained a strong direct effect on reading literacy even after the mediators were included in the model. Therefore, the findings suggested that teacher support influenced reading literacy through multiple pathways, with emotional processes appearing to exert a more prominent role than self-efficacy beliefs during middle childhood.

The first major finding was that perceived teacher support significantly and positively predicted reading literacy. This result suggested that students who perceived higher levels of emotional, academic, and autonomy support from teachers tended to achieve stronger literacy outcomes. The finding was consistent with previous studies that reported positive associations between supportive teacher-student relationships and academic achievement across different educational contexts (Guo et al., 2023; Yang et al., 2021). Within the Chinese Confucian Heritage Culture context, teachers traditionally occupy positions of authority and legitimacy that extend beyond instructional functions alone. Consequently, teacher support may carry greater psychological significance because students often rely on teachers not only for knowledge acquisition but also for validation, encouragement, and academic security (Chen, 2023; Jia and Cheng, 2024). Therefore, the present findings reinforced the importance of supportive teacher-student relationships as a foundational condition for literacy development during primary education.

The second major finding was that achievement emotions significantly mediated the relationship between teacher support and reading literacy. This finding indicated that supportive classroom experiences contributed to stronger literacy outcomes partly because they promoted more positive emotional experiences during learning activities. The result aligned closed with the assumptions of Control-Value Theory, which proposed that students’ emotional experiences are shaped by perceptions of control and task value within learning environments (Pekrun, 2024). When students perceived teachers as supportive, they were more likely to experience enjoyment, confidence, and emotional engagement during reading activities, which subsequently facilitated literacy performance. Previous studies similarly reported that positive achievement emotions enhanced attention, persistence, and cognitive engagement during academic tasks (Hamedi et al., 2020; Dewi, 2020). Although emotional experiences are often overlooked in literacy instruction, the present findings suggested that emotions constituted an important mechanism through which classroom support translated into measurable academic outcomes. Therefore, achievement emotions should be regarded as a central component of literacy development rather than merely a by-product of academic success.

The most theoretically significant finding was that reading self-efficacy did not independently mediate the relationship between teacher support and reading literacy. Although reading self-efficacy was positively associated with both teacher support and reading literacy at the correlational level, its direct effect on reading literacy became non-significant after achievement emotions were included in the model. This finding contrasted with a substantial body of literature that identified self-efficacy as a powerful predictor of academic performance (Bandura, 2005; Bakkaloglu and Pilten, 2023). However, the result may reflect the developmental characteristics of Grade 4 pupils, whose efficacy beliefs remain relatively unstable and highly dependent on immediate contextual experiences. During middle childhood, emotional reactions to classroom events may exert stronger influences on learning behavior than abstract beliefs about personal competence. Consequently, students who felt anxious, discouraged, or disengaged during reading activities may have struggled to translate positive efficacy beliefs into actual literacy performance. Therefore, the findings suggested that emotional experiences may precede and overshadow cognitive-motivational beliefs when students engage in challenging literacy tasks.

The non-significant self-efficacy pathway also contributed to a more nuanced understanding of literacy development within Confucian Heritage Culture contexts. Previous studies conducted in Western educational environments frequently emphasized individual agency, personal confidence, and self-regulated learning as key determinants of academic success. However, educational experiences in CHC contexts are often characterized by stronger teacher authority, greater emphasis on social harmony, and higher sensitivity to interpersonal evaluations. As a result, students may respond more strongly to emotional cues embedded within teacher-student interactions than to internally generated efficacy beliefs. Although further cross-cultural research is required to verify this interpretation, the present findings suggested that emotional mechanisms may represent a particularly influential pathway through which teacher support affects literacy outcomes among Chinese primary school pupils. Therefore, the study extended existing theory by highlighting the potential contextual dependence of psychological mechanisms underlying literacy achievement.

From a practical perspective, the findings offered important implications for literacy instruction and educational leadership. The results suggested that effective literacy teaching should extend beyond the transmission of reading skills and focus equally on creating emotionally supportive classroom environments. Teachers who provided constructive feedback, demonstrated genuine care, encourage student participation, and respected diverse viewpoints were more likely to foster positive emotional experiences that enhanced literacy outcomes. Previous educational reforms often concentrated on curriculum content, instructional strategies, and assessment practices while paying comparatively less attention to students’ emotional experiences. However, the present findings indicated that emotional support may represent a powerful yet underutilized resource for promoting literacy achievement. Therefore, teacher professional development programs should include training in emotional support, classroom climate management, and supportive communication practices.

The findings also suggested that emotional support alone may not be sufficient to strengthen reading self-efficacy. Although students perceived greater confidence when receiving teacher support, this confidence dis not independently translate into improved literacy performance. This result implied that self-efficacy may require additional instructional scaffolding before it can produce measurable academic benefits. Teachers may therefore need to combine emotional support with explicit metacognitive instruction, reading strategy training, and guided practice opportunities. By helping students develop concrete skills for analyzing complex texts, organizing information, and monitoring comprehension, educators may enable students to transform perceived support into sustainable literacy competence. Consequently, future literacy interventions should integrate emotional, cognitive, and strategic components rather than focusing exclusively on motivation or confidence enhancement.

Several limitations should be considered when interpreting the findings. First, the cross-sectional design prevented definitive causal conclusions regarding the relationships among teacher support, achievement emotions, self-efficacy, and reading literacy. Second, several sampling and contextual limitations should be considered when interpreting the findings. Specifically, the sample was drawn exclusively from two public schools in Haidian District, Beijing. Because Haidian District possesses distinctive socioeconomic advantages, highly competitive educational resources, and advanced pedagogical environments compared to other regions in China, the findings may not fully represent primary school students across the country, particularly those in rural, western, or less advantaged educational settings. Consequently, broad claims regarding ‘Chinese pupils’ should be interpreted with caution. Future research should strive to recruit more diverse, nationally representative samples encompassing both urban and rural contexts to verify the generalizability of the proposed model. Third, although reading literacy was measured objectively using a PIRLS-based assessment, the mediating variables were derived from self-report questionnaires and may therefore be subject to social desirability bias. Consequently, the findings should be interpreted with appropriate caution despite the strong statistical support observed in the structural model.

Further research could extend the present study in several important directions. Longitudinal designs would help clarify the developmental trajectories linking teacher support, emotions, self-efficacy, and literacy outcomes over time. Experimental and intervention studies could further establish causal relationships by examining the effectiveness of structured teacher-support programs. In addition, future investigations should incorporate multiple data sources, including teacher ratings, classroom observations, and behavioral indicators of reading engagement. Finally, researchers may consider examining additional contextual variables such as peer support, parental involvement, and socioeconomic status to develop a more comprehensive understanding of literacy development. Through such efforts, future studies may further clarify how supportive educational environments promote both literacy achievement and psychological well-being during primary education.

Based on the findings, this study proposes a perceived teacher support-achievement emotions-self efficacy-literacy (TSEEL) model to explain how teacher support contributes to reading literacy among primary school pupils in China. Drawing upon self-determination theory, control-value theory, and social cognitive theory, the model integrates both direct and indirect pathways through which supportive teaching practices influence literacy development.

The model can be expressed as:

RL=PTS+(αAE)+(βRSE)+ε

Where RL represents reading literacy, PTS represents perceived teacher support, AE represents achievement emotions, RSE represents reading self-efficacy, α denotes the achievement emotions pathway coefficient, *β* denotes the reading self-efficacy pathway coefficient, and ε represents unexplained variance.

The empirical findings suggest that:

α>β

Indicating that achievement emotions exert a stronger influence on reading literacy than reading self-efficacy. Specifically, achievement emotions significantly mediated the relationship between perceived teacher support and reading literacy, whereas reading self-efficacy did not demonstrate an independent mediating effect after achievement emotions were included in the model. This finding suggests that the emotional benefits generated by supportive teacher-student interactions may be more immediately translated into literacy performance than students’ self-perceptions of reading competence.

## 5 Conclusion

This study investigated the relationships among perceived teacher support, achievement emotions, reading self-efficacy, and reading literacy among Grade 4 pupils in Beijing. Using covariance-based structural equation modeling and data collected from 460 students, the study demonstrated that perceived teacher support positively influenced reading literacy through both direct and indirect pathways. Although both achievement emotions and reading self-efficacy were positively associated with teacher support, only achievement emotions emerged as a significant mediator. The findings therefore indicated that emotional experiences played a more influential role than self-efficacy beliefs in translating teacher support into literacy outcomes during middle childhood. Consequently, the study provided empirical evidence supporting the importance of emotional mechanisms within literacy educations.

The findings also extended the application of Self-Determination Theory, Control-Value Theory, and Social Cognitive Theory within the context of Chinese primary education. The significant mediation effect of achievement emotions supported the proposition that supportive learning environments shape academic outcomes by influencing students’ emotional experiences. In contrast, the non-significant self-efficacy pathway suggested that cognitive-motivational beliefs may not always function as the primary mechanism through which teacher support affects literacy performance. Therefore, the study contributed to more differentiated understanding of the psychological processes underlying literacy development and highlighted the potential contextual influence of Confucian Heritage Culture on these mechanisms.

From an educational perspective, the findings emphasized that literacy instruction should not focus exclusively on decoding skills, vocabulary acquisition, or examination performance. Reading classrooms also represent important environments for cultivating positive emotions, academic resilience, and sustained engagement with learning. Teachers who create supportive, inclusive, and emotionally responsive learning environments may significantly enhance students’ literacy development. Consequently, emotional support, constructive feedback, and strategic literacy guidance should be integrated into everyday classroom practice to promote both academic success and lifelong learning capacity among primary school pupils.

## Data Availability

The datasets presented in this study can be found in online repositories. The names of the repository/repositories and accession number(s) can be found in the article/Supplementary material.
