# Supplementary material for: How teacher support shapes reading literacy among urban primary school pupils in China: evidence from achievement emotions and reading self-efficacy
Source: Front Psychol. 2026 Oct 1;17:1909401. doi: 10.3389/fpsyg.2026.1909401 (PMC13668804; doi:10.3389/fpsyg.2026.1909401)
Supplement: Supplementary file 1 [file Data_Sheet_1.PDF]

APPENDIX 1

THE PROGRESS IN INTERNATIONAL READING LITERACY STUDY (PIRLS) 2016 TEST

Enemy Pie

by Derek Munson

illustrated by Tara Calahan King

It was a perfect summer until Jeremy Ross moved in right next door to my best friend Stanley. I did not like Jeremy. He had a party and I wasn't even invited. But my best friend Stanley was.

I never had an enemy until Jeremy moved into the neighborhood. Dad told me that when he was my age, he had enemies, too. But he knew of away to get rid of them.

Dad pulled a worn-out scrap ofpaper from a recipe book.

"Enemy Pie," he said, satisfied.

You may be wondering what exactly is in Enemy Pie. Dad said the recipe was so secret, he couldn't even tell me. I begged him to tell me something—anything.

"I will tell you this, Tom," he said to me. "Enemy Pie is the fastest known way to get rid of enemies.

This got me thinking. What kinds of disgusting things would I put into Enemy Pie?

I brought Dad earthworms and rocks, but he gave them right back.

I went outside to play. All the while, I listened to the sounds of my dad in the kitchen. This could be a great summer after all.

I tried to imagine how horrible Enemy Pie must smell. But I smelled something really good. As far as I could tell, it was coming from our kitchen. I was confused.

I went inside to ask Dad what was wrong. Enemy Pie shouldn't smell this good. But Dad was smart. "If it smelled bad, your enemy would never eat it," he said. I could tell he'd made this pie before.

The oven buzzer rang. Dad put on oven mitts and pulled out the pie. It looked good enough to eat! I was beginning to understand.

But still, I wasn't sure how this Enemy Pie worked. What exactly did it do to enemies? Maybe it made their hair fall out, or their breath stinky. I asked Dad, but he was no help.

While the pie cooled, Dad filled me in on my job.

He whispered. "In order for it to work, you need to spend a day with your enemy. Even worse, you have to be nice to him. It's not easy. But that's the only way that Enemy Pie can work. Are you sure you want to do this?"

Of course I was.

All I had to do was spend one day with Jeremy, then he'd be out of my life. I rode my bike to his house and knocked on the door.

When Jeremy opened the door, he seemed surprised.

"Can you come out and play?" I asked.

He looked confused. "I'll go ask my mom," he said. He came back with his shoes in his hand.

We rode bikes for awhile, then ate lunch. After lunch we went over to my house.

It was strange, but I was having fun with my enemy. I couldn't tell Dad that, since he had worked so hard to make the pie.

We played games until my dad called us for dinner.

Dad had made my favorite food. It was Jeremy's favorite, too! Maybe Jeremy wasn't so bad after all. I was beginning to think that maybe we should forget about Enemy Pie.

"Dad", I said, "It sure is nice having a new friend." I was trying to tell him that Jeremy was no longer my enemy.

But Dad only smiled and nodded. I think he thought I was just pretending. But after dinner, Dad brought out the pie. He dished up three plates and passed one to me and one to Jeremy.

"Wow!" Jeremy said, looking at the pie.

I panicked. I didn't want Jeremy to eat Enemy Pie! He was my friend!

"Don't eat it!" I cried. "It's bad!"

Jeremy's fork stopped before reaching his mouth. He looked at me funny. I felt relieved. I had saved his life.

"If it's so bad," Jeremy asked, "then why has your dad already eaten half of it?"

Sure enough, Dad was eating Enemy Pie.

"Good stuff," Dad mumbled. I sat there watching them eat. Neither one of them was losing any hair! It seemed safe, so I took a tiny taste. It was delicious!

After dessert, Jeremy invited me to come over to his house the next morning.

As for Enemy Pie, I still don't know how to make it. I still wonder if enemies really do hate it or if their hair falls out or their breath turns bad. But I don't know if I'll ever get an answer, because I just lost my best enemy.

Questions:

7. Who is telling the story?

A Jeremy

B Dad

C Stanley

D Tom

8. At the beginning of the story, why did Tom think Jeremy was his enemy?

____

9. Write one ingredient that Tom thought would be in Enemy Pie.

____

10. Why did Tom think it could be a great summer after all?

A He liked playing outside.

B He was excited about Dad,s plan.

C He made a new friend.

D He wanted to taste Enemy Pie.

11. How did Tom feel when he first smelled Enemy Pie? Explain why he felt this way.

12. What did Tom think could happen when his enemy ate Enemy Pie? Write one thing.

13. What were the two things Tom,s dad told Tom to do for Enemy Pie to work?

14. Why did Tom go to Jeremy,s house?
A To invite Jeremy to dinner.
B To ask Jeremy to leave Stanley alone.
C To invite Jeremy to play.
D To ask Jeremy to be his friend.
15. What surprised Tom about the day he spent with Jeremy?

16. At dinner, why did Tom begin to think he and his dad should forget about Enemy Pie?

A Tom did not want to share dessert with Jeremy.
B Tom did not think Enemy Pie would work.
C Tom was beginning to like Jeremy.
D Tom wanted to keep Enemy Pie a secret.
17. How was Tom feeling when Dad passed the piece of Enemy Pie to Jeremy?
A alarmed
B satisfied
C surprised
D confused
18. What was it about Enemy Pie that Dad kept secret?
A It was a normal pie.
B It tasted disgusting.
C It was his favorite food.
D It was a poisonous pie.

19. Look at this sentence from the end of the story: “After dessert, Jeremy invited me to come over to his house the next morning.” What does this suggest about the boys?
A They are still enemies.

B They do not like to play at Tom's house.
C They wanted to eat some more Enemy Pie.
D They might be friends in the future.
20. Use what you have read to explain why Tom's dad really made Enemy Pie.

____

21. What kind ofperson is Tom's dad? Give an example ofwhat he did in the story that shows this.

____

22. What lesson might you learn from this story?

____

QUESTIONNAIRE 2

STUDENTS' PERCEPTIONS OF TEACHER SUPPORT QUESTIONNAIRE (Bru et al., 2010)

Teacher support	Items	Strongly Disagree	Disagree	Agree	Strongly Agree
Teachers' emotional support	PTS_EMO1.I feel the teachers care about me				
	PTS_EMO2.The teachers often praise me				
	PTS_EMO3.The teachers will help me if I have problems				
	PTS_EMO4.The teachers are like my good friends				
	PTS_EMO5.The teachers know what interests I have				
Teachers' autonomy support	PTS_AUT1.I participate in decisions regarding choice of my learning tasks				
	PTS_AUT2.I participate in decisions regarding working methods I shall use				
	PTS_AUT3.I feel I can influence my working situation at school				
Teachers' managerial support	PTS_MAN1.The teachers make sure we do our best in class				
	PTS_MAN2.The teachers make sure we behave well in class				
	PTS_MAN3.The teachers check to see that we do our homework properly				
	PTS_MAN4.When students are disruptive, the teachers are able to handle this				

Teachers' academic support	PTS_ACA1.When we do group work, teachers explain well				
	PTS_ACA2.When we do project work, teachers explain well				
	PTS_ACA3.When we work on our own, teachers explain well				
	PTS_ACA4.The teachers are good at instructing the whole class				

QUESTIONNAIRE 3

ACHIEVEMENT EMOTIONS QUESTIONNAIRE
(Pekrun et al., 2011; Dewi, 2020)

Achievement emotions questionnaire has been designed in order to measure your judgement of your achievement emotions. Please read the items in the questionnaire carefully and make an accurate evaluation of your reading achievement emotions by choosing the number that accurately represents your ability in each item.

	Emotions	Item	Strongly Disagree	Disagree	Neutral	Agree	Strongly Agree
Classroom achievement emotions	AE_CLA1. Enjoyment	I enjoy being in this class					
	AE_CLA2· Hope	I am confident when I attend this class					
	AE_CLA3. Pride	I am proud of myself					
	AE_CLA4· Anger	I am angry					
	AE_CLA5· Anxiety	Thinking about this class makes me feel uneasy					
	AE_CLA6. Shame	I get embarrassed					
	AE_CLA7. Hopelessness	I feel hopeless					
	AE_CLA8. Boredom	I get bored					
	AE_LRN1. Enjoyment	I enjoy acquiring new knowledge					
	AE_LRN2· Hope	I have an optimistic view toward studying					
	AE_LRN3. Pride	I,m proud of my capacity					
	AE_LRN4· Anger	Studying makes me irritated					
	AE_LRN5· Anxiety	I get tense and nervous while studying					
	AE_LRN6. Shame	I feel ashamed that I could't absorb the simplest of details					

	Learning achievement emotions	I feel hopeless when I think about studying					
	AE_LRN8. Boredom	The material bores me to death					
Test achievement emotions	AE_TST1. Enjoyment	For me, the est was challenging and enjoyable					
	AE_TST2. Hope	I have great hope that my abilities would be sufficient					
	AE_TST3. Pride	I'm proud of how well I mastered the test					
	AE_TST4. Relief	I feel very relieved					
	AE_TST5. Anger	I am fairly annoyed					
	AE_TST6. Anxiety	I feel panicky when doing a test					
	AE_TST7. Shame	I feel ashamed					
	AE_TST8. Hopelessness	I have lost all hope that I have the ability to do well in the class					

QUESTIONNAIRE 4

READING SELF-EFFICACY QUESTIONNAIRE
(Koşar et al., 2022)

Reading self-efficacy questionnaire has been designed in order to measure your judgement of your reading self-efficacy. Please read the items in the questionnaire carefully and make an accurate evaluation of your reading self-efficacy by choosing the number that accurately represents your ability in each item.

Items	Strongly Disagree	Disagree	Neutral	Agree	Strongly Agree
RSE1. I can identify the topic of a reading passage.					
RSE2. I can identify the purpose of the author.					
RSE3. I can use my background knowledge about the topic of the reading passage to improve my reading comprehension.					
RSE4. I can find the explicit main idea of a reading passage.					
RSE5. I can find the implied main idea of a reading passage.					
RSE6. I can determine topic sentences in a reading passage.					
RSE7. I can find supporting details in a paragraph.					
RSE8. I can use context clues to guess the meanings of unknown words in a passage.					
RSE9. I can judge whether supporting details are relevant to the topic of the reading passage.					
RSE10. I can distinguish facts from opinions in a reading passage.					
RSE11. I can answer questions on the passage after reading it.					
RSE12. I can use reading strategies like skimming and scanning to enhance my reading comprehension.					
RSE13. I can draw logical conclusions from a reading passage.					

RSE14. I can make logical inferences based on what is given in the reading.					
RSE15. I can take notes of key points as reading a passage.					
